# Shaking up the silence: consequences of HMGN1 antagonizing PRC2 in the Down syndrome brain

**DOI:** 10.1186/s13072-022-00471-6

**Published:** 2022-12-03

**Authors:** Sean J. Farley, Alla Grishok, Ella Zeldich

**Affiliations:** 1 Department of Anatomy and Neurobiology, Boston University Chobanian & Avedisian School of Medicine, Boston, MA USA; 2 Department of Biochemistry, Boston University Chobanian & Avedisian School of Medicine, Boston, MA USA; 3 Boston University Genome Science Institute, Boston University Chobanian & Avedisian School of Medicine, Boston, MA USA

**Keywords:** Epigenetics, Trisomy, Neurodevelopment, Polycomb repressive complex, Chromatin remodeling, Histone modification, Neurodegeneration, Intellectual disability, Nucleosome

## Abstract

Intellectual disability is a well-known hallmark of Down Syndrome (DS) that results from the triplication of the critical region of human chromosome 21 (HSA21). Major studies were conducted in recent years to gain an understanding about the contribution of individual triplicated genes to DS-related brain pathology. Global transcriptomic alterations and widespread changes in the establishment of neural lineages, as well as their differentiation and functional maturity, suggest genome-wide chromatin organization alterations in trisomy. High Mobility Group Nucleosome Binding Domain 1 (HMGN1), expressed from HSA21, is a chromatin remodeling protein that facilitates chromatin decompaction and is associated with acetylated lysine 27 on histone H3 (H3K27ac), a mark correlated with active transcription. Recent studies causatively linked overexpression of HMGN1 in trisomy and the development of DS-associated B cell acute lymphoblastic leukemia (B-ALL). HMGN1 has been shown to antagonize the activity of the Polycomb Repressive Complex 2 (PRC2) and prevent the deposition of histone H3 lysine 27 trimethylation mark (H3K27me3), which is associated with transcriptional repression and gene silencing. However, the possible ramifications of the increased levels of HMGN1 through the derepression of PRC2 target genes on brain cell pathology have not gained attention. In this review, we discuss the functional significance of HMGN1 in brain development and summarize accumulating reports about the essential role of PRC2 in the development of the neural system. Mechanistic understanding of how overexpression of HMGN1 may contribute to aberrant brain cell phenotypes in DS, such as altered proliferation of neural progenitors, abnormal cortical architecture, diminished myelination, neurodegeneration, and Alzheimer’s disease-related pathology in trisomy 21, will facilitate the development of DS therapeutic approaches targeting chromatin.

## Introduction

Intellectual disability is perhaps the most well-known and ubiquitous feature of Down Syndrome (DS), with deficits in intellect as measured by intelligence quotient (IQ) nearly universal among individuals with the condition. The median IQ score in DS is 40, although variability exists and it can range from 10 to 70 [1]. There is often a delay in cognitive development, and individuals tend to have particular deficits in verbal working memory, executive functioning, syntactic processing, and expressive language [2–6].

**Fig. 1:**
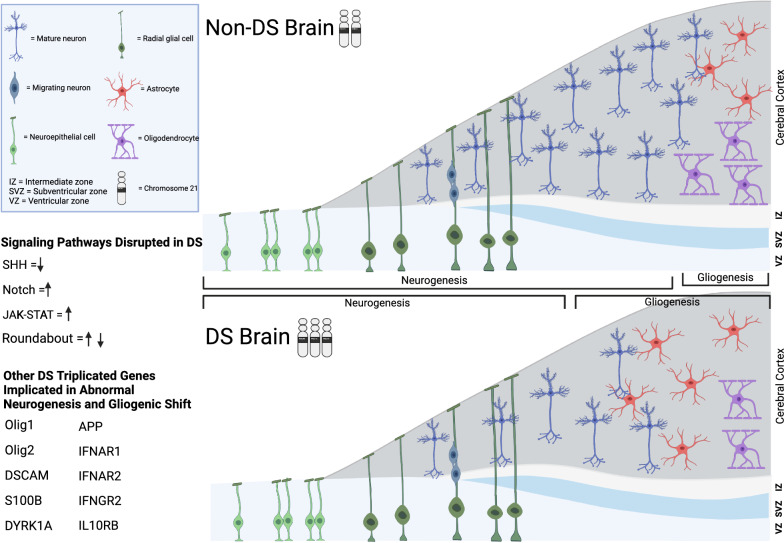
Schematic illustrating the proposed changes in neurogenesis and gliogenesis found in the DS brain. During neurodevelopment, neural progenitor cells, such as radial glial cells, generate neurons that migrate to the cortical plate and populate deep and superficial layers of the neocortex. Neurogenesis then is followed by gliogenesis, which results in the generation of glial cells, such as astrocytes and oligodendrocytes, that subsequently differentiate and mature. A premature shift from neurogenesis to gliogenesis is implicated in DS, with the potential manifestation of impaired neurogenesis, hypocellularity, an increase in the number of astrocytes, and a decrease in the number of mature oligodendrocytes. This precocious gliogenic switch can be attributed to a dosage effect of the triplicated genes (listed in the figure) resulting in a disruption of multiple signaling pathways implicated, shown in the figure. The dysregulation of these pathways can also be a result of the diminished activity of PRC2 due to increased levels of HMGN1 in DS. Created with BioRender.com

Although some treatment or management options exist for other DS-associated conditions, such as congenital heart disease, addressing intellectual disability presents unique challenges since the precise mechanisms underlying the cognitive impairment have been difficult to establish. Many known neurobiological differences in individuals with DS, such as reduced brain volume [7, 8], hypocellularity [9], impairments in cellular proliferation and migration [10], delayed cortical lamination [11], and deficient dendritic arborization and synaptogenesis may contribute to this phenotype [12–14]. In addition, perturbations in glial cells in DS are manifested by precocious gliogenic shift that happens when neural progenitors in the embryonic ventricular zone start to change the fate of the cells they generate from neuronal to glial. This leads to an increased generation of astrocytes and delayed and aberrant myelination [7, 9, 12–16], as schematically shown in Fig. 1. The precise causes of these changes have remained elusive. However, spatiotemporal neurodevelopmental differences in gene expression implicate epigenetic dysregulation in DS as a possible crucial mechanism leading to global transcriptomic alterations and aberrant neural phenotypes.

There are several genes triplicated in DS that affect the epigenetic landscape and may contribute to the described phenotypes, including *High Mobility Group Nucleosome Binding Domain 1* (*HMGN1*), *Dual specificity tyrosine-phosphorylation-regulated-kinase 1A* (*DYRK1A*) [17–21], *ETS proto-oncogene 2* (*ETS2*) [22–30], *Bromodomain and WD repeat domain containing 1* (*BRWD1*) [31–33], *Runt-related transcription factor 1* (*RUNX1*) [34–42], *DNA (cytosine-5)-methyltransferase 3-like* (*DNMT3L*) [43, 44], and *SON RNA and DNA binding protein* (*SON)* [45–50]. This review is focused on *HMGN1*, which is known to promote chromatin decompaction through interactions with nucleosomes [51], and its interplay with the Polycomb Repressive Complex 2 (PRC2) in DS. The disruption of the HMGN1/PRC2 balance is likely to be causatively related to a variety of DS phenotypes. We aim to highlight the consequences of *HMGN1* triplication in connection to the specific transcriptomic, molecular, and cellular changes seen in DS.

### Chromatin architecture and histone modifications

Before we focus our attention on HMGN1 and its functions, we will briefly review some general principles of chromatin compaction.

Genetic information is carried in an incredibly adaptable structure that can be modulated to allow for changes in gene transcription. The repeating structural unit of the human genome is the nucleosome, comprised of DNA wrapped 1.7 times around an octamer structure made of basic proteins called histones. There are two copies of each of the core histone proteins; H2A, H2B, H3, and H4, which form heterodimeric pairs [52]. There are also two linker proteins, H1 and H5, which influence chromatin structure and are known to repress transcription. For an extensive review, please refer to Talbert and Henikoff [53].

Post-translational histone modification via the addition of acetyl, methyl, phosphate, or ubiquitin groups at the N-terminal of histone tails can affect the interaction of these proteins with DNA and lead to alterations in the chromatin structure [54, 55]. This can change how DNA is read and transcribed into RNA. Therefore, histone modifications play an important role in the regulation of the transcriptome.

One important type of histone modification is the addition of an acetyl group to the lysine and arginine residues. Acetylation is present in a majority of N-terminal histone tails and primarily occurs in lysine-rich histone tails (73–80%) versus arginine-rich histone tails (36–48%) [56]. Allfrey et al. [56] demonstrated that the interaction between DNA and histones could be altered by the addition of acetyl groups and that this change in turn impacts the rate of RNA synthesis. Hebbes et al. [57] later showed the presence of acetylated core histones in transcriptionally active genes. This increase in transcriptional activity is likely due to a more open conformation of the nucleosome structure since the addition of an acetyl group to a lysine residue reduces its positive charge, and therefore decreases the strength with which the histone tail interacts with the nucleosomal DNA [58, 59]. This should allow for easier access to the DNA by transcription factors. Indeed, hyperacetylation of H4 is correlated with an elongated shape of the nucleosome core particle [60] and leads to an opening in the tetrameric particle formed by histones H3 and H4, which may allow for easier access to the DNA by transcription factors [61].

Whereas a more relaxed chromatin structure around the histone core following acetylation allows transcription factors to bind to the DNA and activate transcription, the removal of acetyl groups reestablishes the positive charge of the lysine residues and closes the nucleosomal structure, which represses transcription [57, 59, 62]. The addition of acetyl groups is carried out by enzymes known as histone acetyltransferases (HATs) and removed by histone deacetylases (HDACs) [63, 64].

Histones can also be post-translationally modified by the addition of one, two, or three methyl groups to arginine, histidine, or lysine residues. Methylation was originally considered irreversible [65] until the discovery of the demethylases that remove methyl groups [66]. Unlike acetylation, methylation can be either transcriptionally repressive or activating, but most relevant to this review is the transcriptionally-repressive trimethylation of lysine 27 on the histone 3 tail [67, 68]. In addition to directly affecting the properties of chromatin, specific histone modifications can be recognized and bound by the “reader” proteins, which then influence chromatin organization and gene expression. This “histone code” hypothesis, which was postulated by Strahl and Allis [55], has been largely proven by numerous subsequent studies. Importantly, chromatin immunoprecipitation (ChIP) assays using antibodies recognizing specific histone modifications were combined with genomic arrays, and later with deep sequencing, which allowed a generation of genome-wide chromatin modification maps [69–73]. Thus, H3K4, H3K36, and H3K79 methylation has been correlated with transcriptionally active genes [74–78], whereas H3K9, H3K27, and H3K20 methylation is associated with transcriptional repression [67, 68, 79, 80]. Moreover, it was recognized that transcription start sites (TSS) are marked with localized enrichment in H3K4me3 [70, 76, 81, 82], and enhancer elements are marked by H3K4me1 and H3K27ac modifications [73, 83, 84].

A remarkable coincidence of H3K27 methylation domains with smaller H3K4 methylation regions was discovered in mouse embryonic stem cells (ESCs) and termed “bivalent domains” in 2006 [85]. This pattern was later found predominant in human ES cells [86]**.** Sequential ChIP experiments confirmed that these seemingly mutually exclusive modifications co-existed on the same histone H3 tails [85, 86]. Moreover, this bivalent feature was predominantly found on developmental genes activated by lineage-specific transcription factors that were either not expressed or expressed at low levels in ESCs. It was suggested that such genes are poised for future activation [85], and, indeed, the bivalent marks showed dynamic changes during ES cell differentiation [86]. In later sections, we will discuss the relevance of bivalent marking in neurogenesis and DS abnormalities.

To summarize, there are numerous regulators of chromatin organization, and we will focus on the high mobility group (HMG) family of proteins next.

### High mobility group (HMG) proteins

High mobility group (HMG) proteins are key epigenetic regulators found in all vertebrates that exert their effect through chromatin remodeling [87, 88]. They were first extracted from calf thymus tissue and identified as a new class of non-histone proteins by Goodwin et al. [89]. Since then, they have been noted to play a role as global regulators of chromatin architecture and gene expression, mainly by reducing chromatin fiber compaction and promoting DNA accessibility for transcription and replication [51, 90–97], as well as their ability to influence modifications to histone tails [98]. There have been three families of these proteins identified and described: HMGA, HMGB, and HMGN, which are all less than 30 kDa in size and bind reversibly to DNA or nucleosomes [95, 99, 100]. HMGA proteins bind in the minor groove to AT-rich sequences [101], HMGB proteins contain 80 amino acid domains that bind with limited specificity to the minor groove of DNA [102], and HMGN proteins bind to nucleosomes, which are comprised of 146 base pairs of DNA wrapped around a central histone octamer [103]. Digestion of HMGN-associated chromatin with micrococcal nuclease results in DNA fragments that are 10 to 20 base pairs longer than core nucleosome DNA, indicating HMGN may also interact to a limited extent with linker DNA [96].

The HMGN family is made up of five different proteins: HMGN1, HMGN2, HMGN3, HMGN4, and HMGN5 [103–107]. HMGN1 and HMGN2 are expressed most ubiquitously, with HMGN3, HMGN4, and HMGN5 expression limited by tissue type or developmental stage [105, 106, 108–111]. Expression of HMGN3 is found primarily in the eye, brain, and pancreatic islet cells [105]. HMGN4 is expressed in most human tissues, but expression levels are highest in the thyroid gland, thymus, and lymph nodes [107]. This variant is also only found in primates, unlike the others which are found in all vertebrates [112]. The newest member of the family to be discovered, HMGN5, specifically interacts with the linker histone H1 and promotes its chromatin-compacting functions [113].

Similar to other HMGN proteins, HMGN1, an HSA21 triplicated gene, binds within the nucleosome core structure [114]. It has a molecular weight of approximately 10 kDa and contains three main features: a 30 amino acid nucleosome binding domain (NBD) at the N-terminus, nuclear location signals flanking the NBD, and a regulatory domain, i.e. chromatin-unfolding domain (CHUD), at the C-terminus [115]. Within the NBD, there is a sequence of amino acids that reads ‘RRSARLSA’, which confers the protein’s ability to bind to the nucleosome core particle and is a defining feature of the HMGN family [111]. In fact, HMGN proteins are the only non-histone proteins capable of binding between the DNA and the histone octamer core, basically within the nucleosome unit itself [94]. Specifically, HMGN1 binds in the major groove of the nucleosomal DNA near the nucleosome’s dyad axis [94, 103]. Furthermore, the binding of HMGN1 to nucleosomes is regulated post-translationally, occurring only in interphase and being completely abolished during mitosis when two serine residues located at position 20 and 24 in the NBD of HMGN1 are phosphorylated and negate the ability of HMGN to bind nucleosomes [116–118]. This exclusion of HMGN from the nucleosome during mitosis, when DNA is highly condensed, is in line with the proposed role of these proteins in promoting DNA accessibility for transcription and decompacting the chromatin [118].

Here we reviewed the general structure and function of different HMG family members and explained how HMGN1 binds to the nucleosome. Next, we will focus on the effect of HMGN1 on chromatin accessibility for transcription.

### How can the binding of HMGN1 to nucleosomes affect transcription?

Two HMGN1 molecules bind cooperatively to the nucleosome core particle and form a homodimeric complex [119]. A majority of HMGN1 proteins are bound to nucleosomes at any given point, with a mean bind time of 4.1–24.8 s [120].

The first way HMGN1 can alter the structure of chromatin is by affecting linker histone H1’s interaction with the nucleosome since H1 promotes the compaction of chromatin [51, 103, 121]. There is evidence of overlap between the binding of HMGN1 and H1 near the nucleosome’s particle dyad axis [103]. H1 has a higher affinity for the nucleosome, and while quantities of HMGN1 are only sufficient to bind approximately 1% of the nucleosomes in a cell, the movement of HMGN proteins in the nucleus is significantly faster [122, 123]. These competitive dynamics have a direct effect on chromatin compaction, and while loss of HNGN1 enhances H1 binding to nucleosomes [124], increased levels of HMGN1 inhibit the binding of H1, decrease its residence time at nucleosomes [125], and impede its ability to effectively compact the chromatin architecture [51]. Another way HMGN1 antagonizes linker histone H1’s action is by restoring the electrostatic forces that lead to DNA repulsion and cause a more open chromatin conformation [51, 126].

Recent studies demonstrate that the binding of HMGN1 to nucleosomes is further facilitated by the presence of an acetylation mark on H3K27 [123], which enables interactions with different transcription factors that affect gene expression. Zhang et al. [123] performed HMGN1 ChIP-seq analysis in ESCs and demonstrated that it preferentially localizes to nucleosomes containing H3K27ac marks, with no preference for other marks associated with increased transcription, such as H3K9ac and H3K4me1. Moreover, reducing acetylation levels of H3K27 with an acetyltransferase inhibitor resulted in the decreased abundance of HMGN on chromatin. Furthermore, the authors showed that the C-terminal regulatory domain of HMGN1 is not responsible for its preferential co-localization with H3K27ac and therefore cannot be responsible for H3K27ac recognition. This suggests that acetylation of H3K27 promotes nucleosome conformation that allows for easier binding of the HMGN proteins, as well as for their longer time spent bound to the nucleosome. Moreover, in vivo findings from this study using double-knockout (DKO) mice lacking both *HMGN1* and *HMGN2* revealed that the loss of HMGNs resulted in decreased H3K27ac marks [123]. This was accompanied by an increase in H3K27me3 marks and enhanced H1 occupancy, promoting a repressive chromatin state at the enhancer and promoter regions and interfering with the binding of transcription regulators. Furthermore, an additional study found enrichment in the occupancy of HMGN1 and HMGN2 in the areas of super-enhancers, defined as the areas containing a high density of H3K27ac and H3K4me1, in mouse ESCs, MEFs, and resting B cells [127]. In line with this, a very recent report [128] showed that HMGN1 and HMGN2 occupy compartment A (a transcriptionally active nuclear compartment) through their preferential binding to acetylated nucleosomes [128]. While *HMGN1* depletion does not alter the higher-order chromatin structure, HMGNs are specifically localized to the cell-type-specific promoters and enhancers and promote transcription factor accessibility to these open chromatin areas, which form looping interactions, thus contributing to cell type-and stage-distinct gene expression [128].

In summary, HMGN1 competes with histone H1 for the same sites on the nucleosome [87, 125, 129] and preferentially associates with the H3K27ac marks of open chromatin that it helps generate. This allows the binding of transcription factors to enhancers and further leads to a more transcription-permissive conformation of chromatin. The binding of linker histone H1 to the nucleosome is itself transcriptionally repressive, but it also plays a role in the recruitment of Polycomb Repressive Complex 2 (PRC2), which imposes transcriptional silencing [124] as discussed in detail below.

### The biological significance of the polycomb repressive complex 2 (PRC2)

#### PRC2: members, structure, and functions

Gene silencing mediated by PRC2 is believed to be based on the changes in chromatin structure, achieved through post-translational modification of histones. PRC2 is involved in transcriptional silencing through its methyltransferase (HMT) activity, catalyzing the mono-, di-, and tri-methylation of H3K27 [130, 131]. It has many roles specifically within the central nervous system, including effects on maturation [132], proliferation [133], and the identity of neural stem cells [134], migration and maturation of neurons [135], and gliogenesis [136, 137]. The proper function of PRC2 is required for the silencing of specific genes to allow the transcription of other genes that mediate alternative cellular processes.

PRC2 is comprised of three major subunits crucial for this catalytic activity: Embryonic ectoderm development (EED), Suppressor of zeste-12 (SUZ-12), either Enhancer of zeste 1 (EZH1) or EZH2, as well as the histone binding proteins RbAp46 and RbAp48 [18, 138]. EZH1 and EZH2 are proteins encoded by the mammalian homologs of the *E(Z)* gene originally identified in *Drosophila* and appear to have slightly different functions [139]. While both proteins are involved in transcriptional repression, EZH2 exerts its effect primarily as an HMT, whereas EZH1 appears to compact neighboring nucleosomal arrays [138]. The catalytic component of the PRC2 complex, EZH2, adds methyl groups to H3K27, as well as to H3K9, via its SET domain that possesses lysine-specific HMT activity [140]. Both EZH2 and EZH1 interact with the other major subunits of the PRC2 complex, SUZ-12, RbAp46/48, and EED to carry out their functions [138]. EED’s aromatic cage selectively binds to H3K27me3 marks, creating a positive feedback loop where binding to this mark propagates further binding by PRC2 and increases H3K27me3 [141, 142]. The C-terminal region of SUZ-12 is necessary for chromatin binding [143].

The setting of H3K27me3 marks by PRC2 leads to the recruitment of PRC1, an E3 ubiquitin ligase [144, 145] that is believed to silence gene transcription through the interference with RNA polymerase II activity via the mono-ubiquitination of H2AK119 [146, 147]. The crosstalk between PRC1 and PRC2 involves a positive feedback mechanism: PRC1-established H2AK119ub1 marks are recognized by PRC2, while H3K27me3 marks deposited by PRC2 recruit more PRC1 [148]. Recent studies showed that the catalytic activity of PRC1 is essential for the proper functioning of both complexes and gene silencing [149, 150].

#### The role of PRC2 in cell identity acquisition and establishment of neural lineages.

Beyond its global function in transcriptional repression, PRC2 plays specific roles in the process of establishing cellular identity and differentiation. Pereira et al. [134] demonstrated that PRC2 regulates a delicate balance between the self-renewal of cortical progenitor cells and neurogenesis. The researchers used mice carrying “floxed” alleles of *EZH2* crossed with mice from an *Emx1-Cre* line, expressing Cre in the cortical pyramidal neurons [151]. Cre expression was induced starting at embryonic day 9.5 (E9.5) and resulted in the deletion of the EZH2 subunit in cortical progenitor cells. The *EZH2*-KO group showed increased cortical plate volume accompanied by a greater number of neurons, especially of layers V and VI, in the cortex at E14.

By the time of birth, however, *EZH2*-KO animals displayed a thinner cortical plate and fewer cells, as well as the compromised generation of superficial layers’ neurons (II-IV). Using Bromodeoxyuridine (BrdU) pulse labeling of the cortical progenitor cells in vivo at E13, the investigators detected a shift towards the generation of neurons, as well as basal progenitor cells (representing direct and indirect neurogenesis, respectively) at the expense of the self-renewal of cortical progenitor cells that led to premature exhaustion of their pool in *EZH2*-KO group, indicating that neurogenesis is temporally regulated by PRC2.

Similar dynamics were observed when another PRC2 subunit, EED, was deleted from NPCs populating the hippocampal dental gyrus (DG) and the subventricular zone (SVZ) at E13.5 [152]. This was achieved by crossing transgenic *EED* “floxed” mice with mice expressing *Cre* under the *GFAP* promoter. Similarly to the study performed by Pereira et al. [134], there was an initial increase in the number of postmitotic neurons in DG (at P7) followed by a drastic decrease in the numbers at P14 in *EED*-KO animals. This was accompanied by the reduced proliferation capacity of NPCs, suggesting once again that the ablation of the components of PRC2 leads to an impaired pattern of NPC renewal and aberrant neurogenesis.

PRC2 also regulates the acquisition and maintenance of an intact neuronal identity. Conditional deletion of the EED subunit in postmitotic dopaminergic (midbrain DA) and serotonergic (hindbrain 5HT) neurons in transgenic mice led to the diminished transcription of subtype-specific genes and derepression of non-mDA and non-5HT genes, including those coding for transcription factors involved in autoregulatory and fate-determining functions, as well as death-promoting genes [153]. These observations were similar to the finding in medium spiny neurons (MSNs) and Purkinje cells demonstrating that PRC2 activity is essential for shutting down the transcription of the death-promoting genes [154]. EED ablation also resulted in functional deficits manifested in the altered electrophysiological properties of the neurons, abnormal production of cell-specific metabolites, and pathological behavioral phenotypes in mice [153]. In agreement with this, conditional EZH2 inactivation via *Cre*-inducible deletion of the SET domain in glutamatergic neurons in vitro led to their altered differentiation trajectory and the switch towards a transcriptomic signature associated with GABAergic neurons [132]. The enhancement in GABAergic signature and the overproduction of interneurons following EZH2 ablation was also shown in mouse cerebellum [155], further implicating PRC2 in the establishment and maintenance of neuronal identity, as well as the balance between the different neuronal populations.

The proteins of the PRC2 complex also facilitate the appropriate migration of post-mitotic neurons in the cortex during development. The loss of *EZH2* induced through intra-utero electroporation (IUE) of an *EZH2*-specific short hairpin RNA (shRNA) into mouse E14.5 neocortex resulted in abnormal neuronal orientation during the radial migration, with an increase in neurons in the intermediate zone (IZ) and a reduction in those that reached the upper cortical plate (CP) [135]. These findings were further attributed to the ectopically activated expression of *Reelin* in the migrating neurons, suggesting that silencing of *Reelin* in migrating post-mitotic neurons by EZH2 is essential for the proper cortical lamination [135]. Similarly, the ectopic expression of *Netrin 1*, due to the loss of EZH2, results in the abnormal tangential migration of precerebellar neurons in the mouse hindbrain, supporting further the essential role of PRC2-induced gene silencing in neuronal motility [156].

Deviant PRC2 expression can also affect the timing of gliogenesis, and the type of glial cell produced. The onset of gliogenesis in the developing neocortex is tightly regulated and commences at the end of neurogenesis with the production of immature astrocytes [136, 137], followed by the generation of oligodendrocyte precursor cells (OPCs) [157, 158]. Disruption in the timing of gliogenesis in PRC2 mutant mice was demonstrated by Pereira et al. [134] when they found precocious astrocyte development upon *EZH2* KO, with *GFAP*-expressing glial cells found earlier in the cortical plate of an *EZH2* KO group of mice as compared to controls. Overexpression of *EZH2* in mouse embryonic neural stem cells (NSCs), on the other hand, led to a reduction in astrogenesis and an increase in oligodendrocytes [159]. The opposite was seen when the same group used shRNA to reduce *EZH2* expression in mouse embryonic NSCs, with an increase in the percentage of astrocytes and a decrease in oligodendrocytes. Taken together, it is apparent that PRC2 is necessary for proper gliogenesis and balance between astrocyte and oligodendrocyte development.

An increasing body of evidence indicates that oligodendrocyte lineage development is particularly tightly regulated by PRC2, with studies highlighting the necessity of PRC2-mediated repression at different developmental stages ranging from oligodendrocyte lineage commitment to differentiation and maturation. Wang et al. [160] showed that the members of this complex are involved in the switch from the OPC to the differentiated oligodendrocyte stage. This group performed a conditional KO in transgenic mice carrying a floxed *EZH2* allele using an *Oligodendrocyte Transcription Factor 2 (Olig2)-Cre* driver. Olig2 transcription factor governs pMN progenitor domains of the ventral spinal cord that give rise to motor neurons during neurogenesis followed by the production of oligodendrocytes and a subset of astrocytes during gliogenesis [161]. The investigators abolished *EZH2* expression on day E12.5, which resulted in the absence of the H3K27me3 mark in the pMN domain at later time points [160]. No changes were observed in the OPCs produced in the absence of EZH2, indicating that this member of PRC2 is not essential for the generation of OPCs. However, a reduction in the number of *myelin basic protein* (*MBP*)*-*expressing oligodendrocytes by E18.5, suggests that the process of differentiation into mature OLs is delayed when EZH2 is depleted.

Similar results were obtained following a conditional KO of the EED subunit (acting in concert with both EZH1 and EZH2) in *Olig2*-expressing cells. Again, no change was observed in the generation of OPCs, but the number of MBP-positive oligodendrocytes was reduced, and *EED*-KO mice exhibited deficient myelination-associated phenotypes, including aberrant motility, seizures, and tremors, and died by P17. Noticeably, increased production of astrocytes was detected in *EED* KO Olig2-expressing cells, supporting the previous notion that the proteins of the PRC2 regulate the balance between the oligodendrocytes and astrocytes. The orchestrating role of the PRC2 members in the differentiation and maturation of oligodendrocyte lineage was attributed to the repressive control performed by PRC2 on the activation of Notch and Wnt signaling. This study highlights the crucial role of PRC2 in silencing the alternative pathways and allowing the expression of genes leading to functionally mature oligodendrocytes.

High levels of EZH2 are found in proliferating mouse NPCs, followed by decreased expression during the differentiation to neurons and astrocytes in vitro [159]. However, the expression of *EZH2* remains high in NPCs transitioning into OPCs and immature oligodendrocytes. *EZH2* overexpression in NPCs resulted in increased oligodendrocyte production, while silencing of *EZH2* using shRNA led to the enhanced production of neurons and astrocytes.

Douvaras et al. [162] used human ESCs and induced pluripotent stem cell (iPSC) lines to decipher the epigenetic changes that take place during the differentiation of NSCs into OPCs and then oligodendrocytes. The authors detected the enhanced expression of *EZH2* and *EED* mRNAs during the NSC-to-OPC transition and the pre-OPC stages suggesting that the members of PRC2 are involved in the early commitment of NPCs to the OL lineage. This contrasts with the study performed by Wang et al. [160] highlighting the requirement for the EZH2 activity at later stages of oligodendrocyte development, but still implicates PRC2 in this process. Importantly, ESCs showed consistently increasing H3K27me3 levels during the transition from the NSC stage all the way to immature oligodendrocytes, reflecting again the importance of this mark in oligodendrocyte differentiation. Interestingly, EZH1 was most highly expressed in immature oligodendrocytes, suggesting EZH1’s role in gene silencing once oligodendrocyte commitment has occurred. Taken together, this data supports the regulatory role of PRC2 in oligodendrogenesis, oligodendrocyte differentiation, and maturation.

The only study connecting PRC2-mediated control of oligodendrocytes differentiation and *HMGN1*, the main focus of this review, was performed by Deng et al. [124]. This work showed that the interplay between HMGN1 and PRC2 impacts *Olig1* and *Olig2* expression. ChIP-qPCR analysis revealed an increase in the levels of H3K27me3, as well as the EZH2 subunit of the PRC2, in the genomic locus containing *Olig1* and *Olig2* in *HMGN*^−/−^ ESCs. Further, they showed an increase in *Olig1* and *Olig2* expression in vitro in ESCs that were treated with an EZH2 inhibitor, GSK126. The authors postulated that the absence of HMGN1 increased the ability of linker histone H1 to bind to the nucleosome, promoting deposition of the transcriptionally repressive mark H3K27me3 by PRC2 and reducing the expression of *Olig1* and *Olig2* [124]. This is an interesting finding since *Olig1*, *Olig2*, and *HMGN1* are all triplicated in DS. It raises the question of how increased levels of HMGN1 further impact the levels of *Olig1* and *Olig2* and influence oligodendrocyte development in trisomy.

In this section, we reviewed literature reflecting the crucial role of PRC2 and its different subunits in various neurodevelopmental processes. Research to date demonstrates that PRC2 activity is essential for the maintenance of balance between the self-renewal of the cortical progenitors and their differentiation into neurons, establishment of cell identity and neuronal migration, as well as for the timely transition from neurogenesis to gliogenesis and proper differentiation and maturation of oligodendrocytes. We summarize the developmental effects of PRC2 in Table 1.

**Table 1:** Developmental effects of PRC2

Developmental effect	Related findings	References
Proper timing of neurogenesis	EZH2-KO in mice leads to premature exhaustion of the pool of neural progenitor cells, decreased neuronal density at the cortical plate, and precocious astrocyte generation	Pereira et al. [134]
	EED-KO mice experience impaired neurogenesis, growth retardation, and death. EED ablation leads to abnormal neuronal differentiation during the hippocampal dentate gyrus formation	Liu et al. [152]
	miR-203 is repressed by EZH2 in neural progenitor cells and negatively regulates proliferation. EZH2-KO results in the reduction of neural progenitor proliferation	Liu et al. [166]
Appropriate neuronal orientation and cortical radial migration	EZH2 inhibition results in abnormal neuronal orientation reduced neuronal numbers at the cortical plate and ectopic expression of Reelin	Zhao et al. [135]
	EZH2-mediated repression of *Netrin1* is necessary for the appropriate migration of pontine neurons in the cortico-ponto-cerebellar pathway in mice. EZH2-KO results in ectopic *Netrin1* induction and aberrant neuronal migration	Di Meglio et al. [156]
Maintenance of neuronal identity	Deletion of EED disrupts the acquisition and maintenance of neuronal identity and functionality in differentiated dopaminergic and serotonergic neurons	Toskas et al. [153]
	Conditional knock-out of EZH2 results in reduced proliferation of granule precursor cells, decrease in the Purkinje cell population and increase in GABAergic interneurons in the mouse cerebellum	Feng et al. [155]
	Conditional KO of EZH2 results in loss of H3K27me3 marks in differentiating neurons and causes changes to molecular networks that govern glutamatergic neuron differentiation, leading to a disruption in the balance of inhibitory/excitatory neurons during the development	Buontempo et al. [132]
	Combined EZH1 and EZH2 KO leads to a loss of H3K27me3 marks in MSNs in the striatum, down-regulation of lineage-specific and function-specific MSN genes, and upregulation of the death-promoting genes	Von Schimmelmann et al. [154]
Glial cell development and fate determination	Pharmacological inhibition of EZH2 leads to increased expression of Olig1 and Olig2. Increased depositions of H3K27me3 marks are detected in the genomic loci of *Olig1* and *Olig2* in HMGN-KO ESCs	Deng et al. [124]
	KO of EZH2 or EED do not affect the generation of OPC but inhibit their differentiation into mature, myelinating oligodendrocytes. PRC2 is necessary for the repression of the Notch pathway	Wang et al. [160]
	EZH2 regulates NSC differentiation into glial cells in mice, with high expression levels of EZH2 associated with increased oligodendrocyte production and decreased production of astrocytes while low levels of EZH2 correlate with a reduction in oligodendrocyte generation and increased numbers of astrocytes	Sher et al. [159]
	Increased levels of *EZH2* and *EED* mRNAs are detected during the early stage of OPC lineage commitment and development in mouse and human ESCs and human iPSCs	Douvaras et al. [162]

Summary of studies demonstrating the role of PRC2 in neurodevelopment

#### Bivalent marks and their significance in neural development

As was discussed earlier, genes activated during differentiation are marked by both “silencing”, H3K27me3, and “activating”, H3K4me3 marks in undifferentiated ESCs. Given the critical role of PRC2 in NPC differentiation, it is important to review recent insights into bivalent chromatin dynamics during this process. Importantly, bivalency is not limited to ESCs and is present in other progenitor populations defined as multipotent, such as NPCs and glial progenitors [144, 163–165]. Liu et al. [166] compared genome-wide RNA Polymerase II (Pol II), H3K4me3, and H3K27me3 landscapes between purified mouse NPCs and neurons. Remarkably, the authors found that bivalently marked genes in NPCs included those critical for cortical neuron differentiation, migration, and function. These genes acquired more prominent H3K4me3 marking in neurons, consistent with their higher expression. At the same time, genes known to express highly in OPCs, such as *Olig1, Olig2,* and *platelet-derived growth factor receptor alpha *(*Pdgfr-α)* were also bivalently marked in NPCs but got enriched in H3K27me3 in neurons, consistent with their repression in this lineage. The interplay between HMGN1 and PRC2 in *Olig1* and *Olig2* regulation shown in ESCs and discussed above, and data from the animal-extracted cells described by Liu et al. [166], strongly suggest HMGN1’s involvement in the regulation of bivalent genes during NPC differentiation in vivo.

Another important study by Yu et al. used a novel approach to define bivalent genes based on a quantitative assessment of chromatin compaction [167]. The authors purified and sequenced DNA associated with mono-nucleosomes after the treatment of chromatin with Micrococcal nuclease (MNase). They used the “time-course digestion with reduced MNase levels followed by high-throughput sequencing” (TC-rMNase-seq) approach for separately sequencing DNA fragments originating from the open chromatin regions and those coming from more compacted areas. In this manner, they defined a specific moderately compacted chromatin state, which was associated with bivalent genes present in mouse ESCs. Moreover, the authors noted several genes important for neurogenesis among those in the moderately compacted group and followed chromatin changes at these loci upon mESC differentiation into NPCs. Indeed, promoters of these genes showed higher susceptibility to MNase digest in NPCs compared to mESCs in the TC-rMNase-seq assay, consistent with their higher expression in NPCs and accumulation of higher levels of H3K4me3.

The new insight connecting moderate chromatin compaction to bivalency and its resolution during differentiation also supports the proposed role of HMGN1 in opposing PRC2 via its chromatin-opening function.

#### PRC2 and neurodegeneration

PRC2 deficiency has been linked to neurodegeneration in several studies [168–170]. A study performed by von Schimmelmann et al. [154], determined that PRC2 inhibition in adult neurons in vivo leads to an upregulation of death-promoting genes in MSNs in the striatum. PRC2 inactivation was achieved by the combination of a null mutation in *EZH1* and the conditional ablation of the *EZH2* gene in the *Calcium/Calmodulin Dependent Protein Kinase II Alpha (Camk2a)-*expressing neurons marking MSNs. Most of the upregulated genes detected at three and 6 months post-PRC2 ablation were identified as H3K27me3 targets, suggesting a specific regulatory role of PRC2 in adult neurons. Interestingly, the investigators pointed out redundancy in the activity of EZH1 and EZH2 in adult postmitotic MSNs, while previous studies showed that EZH1 is incompetent to substitute for the lost activity of EZH2 in dividing cells during neurodevelopment [138]. By using conditional ablation of the PRC2 members in adult neurons, the researchers deduced that PRC2 deficiency led to the downregulation of MSN-specific genes and the up-regulation of death-promoting genes previously identified as PRC2 target genes, such as *Phorbol-12-Myristate-13-Acetate-Induced Protein 1* (*Pmaip1*), *BH3 Interacting Domain Death Agonist* (*BID*), *NADPH Oxidase Activator 1* (*Noxa*), *Cyclin-dependent kinase inhibitor 2A/B* (*Cdkn2a/b*), and *Insulin-like growth factor-binding protein 3* (*Igfbp3*). This was accompanied by the phenotypic signs of neurodegeneration manifested in the elevation of gamma-H2Ax, a marker of DNA damage, cytoplasmic and nuclear condensation, a decrease in the number of MSNs, and a reduction in the total brain mass.

Furthermore, altered PRC2 activity in MSNs manifested in neurodegenerative phenotypes and behavior changes, as mutant mice showed impairments in rotarod performance, difficulty in hanging on a wire top, and altered hind limb clasping followed by the termination of all voluntary eating and drinking and death at 7 months. The same approach was taken to abolish the activity of PRC2 in Purkinje cells (PC) in the cerebellum. The loss of PRC2 in PC resulted in the upregulation of death-promoting PRC2 target genes and progressive neurodegenerative phenotypes accompanied by abnormal motor behavior phenotypes [154]. Notably, the authors concluded that PRC2 silencing of the bivalent genes in particular is essential for the maintenance of cell identity in adult neurons. The upregulation of the pro-apoptotic genes was also detected in differentiated serotonergic and dopaminergic neurons upon EED deletion but did not result in the induction of cell death [153]. Altogether, these studies strongly implicate PRC2 activity in the repression of neurodegeneration-related pathways thus ensuring the survival of adult neurons [153, 168–170].

### Implications of HMGN1 gene dosage effect on PRC2-mediated gene silencing in DS

The first possible link between DS and PRC2 was established by a group studying B-cell acute lymphoblastic leukemia (B-ALL) [171]. Individuals with DS are at a 20-fold risk for developing B-ALL compared to the general population [172] and 60% of B-ALL cases in DS harbor the rearrangement of cytokine receptor-like factor 2 (*CRLF2*) further classifying it as DS-ALL [173]. Lane et al. [171], aimed to determine what confers this increased risk using Ts1Rhr mice harboring a triplicated fragment of the mouse chromosome 16 (Mmu16) orthologous to a human chr.21q22 segment that includes *HMGN1* among other 31 genes on this fragment. Ts1Rhr mice consistently developed B-ALL, and the progenitor B cells derived from the bone marrow of these mice showed an enhanced formation of colonies and increased rate of cell renewal as compared to WT, supporting a transformed phenotype of these cells.

Comparison of the transcriptomic signature of Ts1Rhr mice against human DS datasets from different cohorts done through the network enrichment analysis identified the PRC2 target genes and the H3K27me3 sites as the most highly enriched. Furthermore, differential expression of PRC2 and H3K27me3-target genes was sufficient to differentiate between DS-ALLs and other B-ALLs. Importantly, genes defined as PRC2 targets were all found to be upregulated in DS-ALL, suggesting that increased transcription and reduced silencing of PRC2 targets in DS-ALL were the root cause of these differences. Further support for this assertion was provided by mass spectrometry of H3 showing that the Ts1Rhr B-cells had a reduction in H3K27me3 peptides, and ChIP analysis revealing a global reduction in H3K27me3 marks. This data obtained from the Ts1Rhr mice provided evidence that the triplication of 31 genes orthologous to the human DS-critical region was sufficient to reduce H3K27me3 occupancy.

Remarkably, overexpression of HMGN1 alone in vivo was sufficient to reproduce many of the transcriptional and phenotypical features seen in the Ts1Rhr B cells with all 31 genes triplicated. These results support the crucial role of HMGN1 in the de-repression of the PRC2 target genes in DS. HMGN1 overexpression was further implicated in transcriptomic changes and histone modifications related to DS-ALLs in a subsequent study [174] that revealed a global increase in RNA transcripts produced per gene in pro-B cells from the Ts1Rhr cells as compared to the WT ones. Using transgenic mice overexpressing *HMGN1* (*HMGN1-OE*) the investigators showed a genome-wide transcriptional amplification, accompanied by the global increase in H3K27ac marks in *HMGN1-OE* cells, similar to the Ts1Rhr cells. This supports the idea that HMGN1 is responsible for a global increase in gene expression in DS. Further gene set enrichment analysis showed that genes with enhanced expression induced by *HMGN1-OE* in B cell progenitors were enriched for EZH2 targets.

Remarkably, using B cell lines carrying a doxycycline-inducible *HMGN1* cassette with either WT or mutated nucleosome-binding domain showed that the binding of HMGN1 to the nucleosome was essential for the enhancement of gene expression [174]. Moreover, genes showing a greater increase in expression following the induction of *HMGN1-OE* also displayed the enrichment in H3K27ac at the super-enhancer regulatory regions. Finally, normalizing the expression of only the *HMGN1* gene in the Ts1Rhr mouse DS model rescued B cell pathological phenotypes, abolished an increase in H3K27ac marks, and mitigated mRNA expression changes, indicating that the increased dosage of *HMGN1* is sufficient to induce DS-specific transcriptomic and epigenetic signatures and cellular phenotypes in B cells.

The most recent study applied *HMGN1* CRISPR/Cas9 KO to the SET2 cell line edited to harbor the rearrangement of *CRLF2* observed in DS-ALL patients and to the DS-ALL xenograft mouse model [175]. The authors showed that KO of *HMGN1* abrogated the abnormal proliferation of the SET2 cells in vitro as well as enhanced survival of the DS-ALL xenograft model through mitigation of its disease phenotypes. Using the complementary approach of *HMGN1-OE*, the study concluded that *HMGN1* facilitates leukemic transformation to DS-ALL due to its ability to generate a positive feedback mechanism resulting in the upregulated transcription of *CRLF2* and dysregulation of the downstream signaling pathways and highlighted the crucial role of *HMGN1* in DS-specific ALL.

Further support for the global genomic and epigenetic disruption in trisomy comes from a recent study conducted by Meharena et al. [176]. The authors found evidence for the disorganization in the nuclear architecture and alterations in the transcriptomic signature of human NPCs derived from DS iPSC lines compared to isogenic controls. They demonstrated an increase in H3K27ac and a decrease in H3K27me3 marks in trisomic NPCs, but not in trisomic iPSCs, which suggests developmental and stage-specific alterations of these marks in trisomy. Furthermore, transposase-accessible chromatin sequencing (ATAC-seq) showed a significant increase in differentially accessible regions caused by trisomy in NPCs compared to iPSCs (20% versus 1.6%, respectively). Consistent with this, the transcriptomic profile of trisomic NPCs showed a decrease in *EZH2* and *EED* mRNA, as well as some histone acetyltransferases, further supporting the proposed diminished activity of PRC2 in DS, possibly due to increased dosage of *HMGN1*. Remarkably, transcriptional profiling of trisomic NPCs revealed senescence-associated signatures as well as upregulation of genes related to cell migration, adhesion, and inflammation. This suggests the disturbance of distinct signaling pathways due to enhanced transcription of specific genes in DS can be potentially attributed to the gene dosage effect of *HMGN1*. The concept that increased gene dosage of HMGN1 is necessary and sufficient for the induction of DS-specific ALL suggests the possibility that *HMGN1* triplication can be detrimental in other DS-related phenotypes observed in different systems. The existing studies addressing the triplication of *HMGN1* in DS are summarized in Table 2.

**Table 2:** HMGN1 and DS

DS Phenotype	Title	Related findings	References
Overexpression of HMGN1	Chromosomal protein HMG-14 gene maps to the Down syndrome region of human chromosome 21 and is overexpressed in mouse trisomy 16	Ts16 mouse model of DS has 1.5 times more HMGN1 protein and mRNA than WT and supports the gene dosage effect of HMGN1 triplication	Pash et al. [192]
	Chromosomal protein HMG-14 is overexpressed in Down syndrome	Fibroblasts from DS individuals express HMGN1 at levels 1.6 higher than non-DS individuals	Pash et al. [193]
	Functional transcriptome analysis of the postnatal brain of the Ts1Cje mouse model for Down syndrome reveals global disruption of interferon-related molecular networks	HMGN1 is upregulated in the Ts1Cje mouse model of DS	Ling et al. [194]
	Transcriptional disruptions in Down syndrome: a case study in the Ts1Cje mouse cerebellum during post-natal development	HMGN1 was overexpressed in the cerebellum of the Ts1Cje mouse model of DS during post-natal development	Potier et al. [195]
	Bioinformatics analysis of biomarkers and transcriptional factor motifs in Down syndrome	Raw gene expression data from DS rat brain tissue, Ts1Cje cerebellum tissue, and adult human DS tissue analyzed using Gene Expression Omnibus and showed overexpression of HMGN1	Kong et al. [201]
	Down syndrome developmental brain transcriptome reveals defective oligodendrocyte differentiation and myelination	Raw gene expression data analyzed in humans with DS using Gene Expression Omnibus and found HMGN1 overexpressed in the hippocampus, cerebellar cortex, and areas of the pre-frontal cortex, primary visual cortex,	Rodriguez-Ortiz et al. [203]
Intellectual disability	Genetic contributions to variation in general cognitive function: a meta-analysis of genome-wide association studies in the CHARGE consortium	A genome-wide association study of general cognitive function in adults found HMGN1 as the single gene-based significant association in the study	Davies et al. [288]
Cancer	Triplication of a 21q22 region contributes to B cell transformation through HMGN1 overexpression and loss of histone H3 Lys27 trimethylation	HMGN1 suppresses H3K27me3 and promotes B cell proliferation in B-ALL	Lane et al. [171]
	Trisomy of a down syndrome critical region globally amplifies transcription via HMGN1 overexpression	HMGN1 globally amplifies transcription	Mowery et al. [174]
	HMGN1 plays a significant role in CRLF2-driven Down Syndrome leukemia and provides a potential therapeutic target in this high-risk cohort	HMGN1 is involved in signaling pathways in CRLF2-driven DS leukemia	Page et al. [175]
Behavioral changes	The chromatin-binding protein HMGN1 regulates the expression of methyl CpG-binding protein 2 (MECP2) and affects the behavior of mice	HMGN1 downregulates MeCP2 and its aberrant expression produces behavioral changes in mice consistent with ASD and DS phenotypes	Abuhatzira et al. [180]

Summary of studies associating HMGN1 expression and changes found in DS

### The gene regulatory function of HMGN1: how specific is it to neural development?

While HMGN proteins exert a global effect on chromatin compaction, an increasing body of evidence suggests that *HMGN1* expression is tightly involved in developmental regulation. Since this review is targeting the potential role of *HMGN1* in DS brain pathology, in this section we will be focusing on the role of *HMGN1* in CNS development, which we also summarize in Table 3.

**Table 3:** Role of HMGN1 in CNS Development

Title	Major findings	References
Developmental role of HMGN proteins in *Xenopus laevis*	Altered HMGN1 levels lead to malformations in *Xenopus laevis* development at the post-blastula stage	Körner et al. [178]
High-mobility group proteins 14 and 17 maintain the timing of early embryonic development in the mouse	HMGN1 protein is necessary for the appropriate timing of embryo development in mice; depletion leads to developmental delays	Mohamed et al. [177]
Binding of HMGN proteins to cell specific enhancers stabilizes cell identity	Loss of HMGN1 protein accelerates reprogramming of MEFs into iPSCs	He et al. [127]
High mobility group nucleosome-binding family proteins promote astrocyte differentiation of neural precursor cells	*HMGN1* expression promotes astrocyte differentiation	Nagao et al. [179]
HMGN1 modulates nucleosome occupancy and DNase I hypersensitivity at the CpG island promoters of embryonic stem cells	Loss of *HMGN1* reduces the number of Nestin-positive NPCs in SVZ in mouse brain	Deng et al. [129]
Interplay between H1 and HMGN epigenetically regulates OLIG1 and 2 expression and oligodendrocyte differentiation	Loss of *HMGN1* reduces *OLIG1* and *OLIG2* expression and impairs normal oligodendrocyte differentiation Loss of HMGN1 decreases the amount of MBP and proteolipid protein (PLP) in the spinal cord of mice	Deng et al. [124]

Summary of studies demonstrating the importance of HMGN1 in CNS development

Early embryonic development in mice is disrupted as early as the blastocyst stage by depleted levels of HMGN1 achieved via injection of antisense oligonucleotides [177]. Studies in post-blastula *X. laevis* embryos exposed to microinjection of the HMGN1 recombinant protein revealed that increased HMGN1 levels resulted in profound developmental abnormalities, such as impaired blastopore closure and an inappropriate body axis establishment accompanied by aberrant head structures [178]. *HMGN1* is expressed in the cells of neural lineage: it is detected as early as E12.5 and is highly expressed in the developing E18.5 mouse neocortex, in forebrain VZ and SVZ and cortical plate as well as in the postnatal NPCs and glial progenitors (P7) and some expression of *HMGN1* persists in adult brains in astrocytes and to the lesser extent in postmitotic neurons [179]. Deng et al. [129] investigated the particular role of HMGN1 in neural development using ESC derived from the *HMGN1-KO* transgenic mouse model. In vitro differentiation of ESCs into dopaminergic neurons did not result in noticeable phenotypic alterations but was accompanied by a transcriptomic dysregulation of genes involved in organ morphogenesis, vasculature development, and tissue development. This was detected at the stages of ESCs, neural progenitor cells (NPCs), and differentiated neurons. The same study showed that in vivo HMGN1 is strongly expressed in the SVZ of two weeks old wild type mice and is co-expressed with Nestin, a marker of NPCs. *HMGN1*-*KO* mice, however, displayed a decrease in the number of Nestin-positive cells and *Nestin* mRNA expression in the SVZ compared to the wild type, suggesting the regulatory role of HMGN1 in the maintenance of the NPC’s pool and further neocortical development. This study also demonstrated that in ESCs and NPCs the HMGN1 protein preferentially binds to TSS of active promoters containing CpG islands and regulates the stability and positioning of nucleosomes in these regions, further supporting the role of *HMGN1* in transcriptional gene regulation during development.

A more recent study [127] demonstrated that HMGNs stabilize the epigenetic landscape in specific cell types and allow for the proper establishment and maintenance of cell identity through their clustering at the areas of super-enhancers. The researchers utilized MEFs, ESCs, and neurons derived from *HMGN1* and *HMGN2* DKO mice and observed enhanced efficiency of pluripotency reprogramming during the process of transformation of MEF cells into iPSCs using Yamanaka factors. They also found that further differentiation of ESCs into neurons was accelerated in the absence of both *HMGN1* and *HMGN2*. This suggests that their loss results in reduced accessibility of enhancer regions to the transcription factors maintaining cell type specificity, while their presence stabilizes cell identity. HMGN1 not only regulates the differentiation of NPCs into neurons but is also involved in the switch from neurogenesis to gliogenesis and the generation of astrocytes and oligodendrocytes. *HMGN1* overexpression in mouse forebrain NPCs in vitro resulted in enhanced production of astrocytes and decreased production of neurons, but no change in the generation of oligodendrocytes [179]. Conversely, the knock-down of *HMGN1* expression resulted in a decrease in the production of astrocytes and an increase in the fraction of neurons. Similar results were observed in vivo when *HMGN1* was overexpressed through IUE into the fetal mouse neocortical NPCs. This led to the greater numbers of astrocytes accompanied by a decreased generation of superficial layer neurons at P7 suggesting that HMGN1 regulates the generation of astrocytes in vitro and at perinatal stages in vivo. Remarkably, the knockdown of the *HMGN1* expression postnatally (at P0), decreased the fraction of cortical astrocytes at P7 and led to the over-production of the immature neurons. Together, these findings indicate that HMGN1 plays a role in astrocyte differentiation prenatally as well as postnatally in vivo.

HMGN1 has also been implicated in oligodendrocyte development. Deng et al. [124] differentiated ESCs derived from *HMGN1* and *HMGN2* DKO mice into embryoid bodies (EBs) and towards the oligodendrocyte lineage and observed a decrease in the expression of *Olig1* and *Olig2* transcription factors throughout the process. A decrease in Olig2-positive cells was also shown in vivo in the spinal cord of the DKO mice and was accompanied by the decreased expression of oligodendrocyte lineage markers across the differentiation stages. Specifically, PDGFR-α was downregulated in the OPCs, and 2ʹ,3ʹ-cyclic-nucleotide 3ʹ-phosphodiesterase (CNPase), proteolipid protein (PLP), and MBP were downregulated in the more mature oligodendrocytes. These results implicate the HMGN proteins in the generation, differentiation, and maturation of oligodendrocytes.

Taken together, the existing studies strongly support the regulatory role of HMGN1 in the CNS starting from the early embryonic development and continuing through the later stages of CNS development and cell maturation. HMGN1 has been shown to stabilize the cell identity of the neural lineages and to play an important role in the maintenance of the pool of NPCs and controlling their differentiation into neurons, astrocytes, and oligodendrocytes.

### HMGN1 and changes in behavior

HMGN1 dysregulation may lead to various developmental abnormalities beyond DS through its global epigenetic regulatory function and subsequent downstream effects on the expression of other genes. Abuhatzira et al. [180] showed for the first time that altered HMGN1 levels in mice lead to behavioral changes and autism-related features. This group also showed that HMGN1 controls the expression of *methyl CpG-binding protein 2* (*MeCP2*), dysregulation of which has been linked to the behavioral patterns associated with autism spectrum disorder (ASD) [181]. Abnormal *MeCP2* expression is correlated with cognitive disabilities [181], and transgenic mice expressing 50% reduced levels of MeCP2 demonstrated significant impairments in learning and memory tasks [182].

MeCP2 is thought to participate in transcriptional repression due to its preferential association with methylated DNA [183] as well as with histones carrying the H3K27me3 mark via its methylation binding domain (MBD) [184–187]. Similar to the action of the linker histone H1, MeCP2 can induce compaction of the chromatin fiber and promote transcriptional repression [184]. Reducing H3K27me3 with GSK23, an EZH2-specific inhibitor, results in a decrease in MeCP2 levels on chromatin, as determined by ChIP-seq analysis, indicating that the recruitment of MeCP2 to genomic loci is dependent on H3K27me3 [186]. This is further supported by the co-localization of MeCP2 and H3K27me3 at TSS [183, 186] resulting in a cooperative regulation of transcriptional repression.

Abuhatzira et al. [180] showed that HMGN1 binds to the promoter and the first exon of *MeCP2* in the human and mouse brain cells and regulates its expression. In line with this, in DS human brains, the 50% increase in transcript levels of *HMGN1* is inversely correlated with *MeCP2* transcript levels, which are decreased by 30%. This group also showed that overexpression of *HMGN1 *in vivo or in vitro using MEFs from *HMGN1-KO* mice transfected with an *HMGN1-OE* vector resulted in a decrease in mRNA and protein levels of MeCP2, while KO of *HMGN1* led to a significant increase in the mRNA and protein levels of MeCP2 [180]. The most profound findings of this work led to the discovery that changes in the level of HMGN1 can lead to autism-related behaviors. Through the battery of behavioral tests to evaluate social memory and preference, the study showed that both groups with either upregulation or downregulation of HMGN1 demonstrated a lack of preference in a novel social interaction and novel object recognition, behaviors linked to autistic-like phenotypes [188] and DS-associated cognitive deficits [189]. Notably, this work did not show that the behavioral changes induced by misexpression of *HMGN1* were mediated by direct changes to *MeCP2* but instead recognized the global effect of HMGN1 expression on chromatin accessibility. It is possible, however, that reduced *MeCP2* expression due to the high levels of HMGN1 in trisomy can potentially promote further decompaction of the chromatin.

To the best of our knowledge, the study described above is the only investigation directly showing that altered levels of HMGN1 are causatively linked to the behavioral features associated with the autistic and DS-related phenotypes.

### HMGN1 expression in mouse and human DS brain tissue

DS is caused by the presence of all or part of an extra copy of chromosome 21 [190], and the region of q22.1 to q22.3 is considered to be critical for DS [188, 191]. The *HMGN1* gene was mapped to a 21q22.3 region of human chromosome 21 by Pash et al. [192, 193]. The gene dosage effect of *HMGN1* was observed in several mouse models of DS. The Ts16 mouse model (harboring the triplication of Mmu16) shows a 1.6- to 3.3-fold increase in the *HMGN1* mRNA in the brain and whole trunk of Ts16 fetuses, respectively [192]. The *HMGN1* mRNA was found to be increased in the cortex, hippocampus, and cerebellum of the Ts1Cje mice [194, 195], representing another mouse model of DS that contains a partial triplication of Mmu16 [196], and in the embryonic brain tissue of Ts65Dn [197], the most widely used mouse model of DS, containing an extra-small chromosome resulting from a fusion of the region of Mmu16 orthologous to Hsa21 with the centromeric region of Mmu17 as well as the additional extra segment of non-DS-related genes [198]. Increased hippocampal *HMGN1* mRNA expression in adult mice has also been confirmed in the Dp1Tyb mouse model that contains an additional copy of 63% of mouse genes orthologous to the HSA21 genes [199]. The *HMGN1* mRNA levels are also increased in the forebrain tissue at postnatal day 1 in a novel non-mosaic DS model, TcMAC21, which incorporates 93% of HSA21q protein coding genes as a segregated chromosome [200]. The correlation between the gene dosage imbalance and the *HMGN1* expression upregulation across different DS mouse models is of extreme importance since a large part of our understanding of DS pathology and the underlying molecular mechanisms are based on the high utility of mouse models of trisomy 21.

Importantly, the bioinformatic analysis of the data obtained through Gene Expression Omnibus (GEO) concluded that *HMGN1* is at least 1.5-fold upregulated in the human trisomic tissue in an orthologous comparison to the mouse trisomic tissue [201]. Human cultured DS fibroblasts also exhibit a 1.6-fold increase in *HMGN1* mRNA expression, and increased protein levels of HMGN1 are detected in human embryonic DS brains compared to control samples [192, 193]. In addition, *HMGN1* mRNA expression is upregulated in the cerebellum and dorsal prefrontal cortex across multiple developmental periods in DS human postmortem brain tissue compared to controls [202].

Rodriguez-Ortiz et al. [203] conducted a study to determine the distribution of *HMGN1* expression in the brain of individuals with DS. The authors accessed raw gene expression data collected from individuals with and without DS from GEO, focusing on a microarray experiment that compared gene expression in multiple brain areas. They found that *HMGN1* is overexpressed in DS brains compared to control in the hippocampus, cerebellar cortex, and primary visual cortex, as well as areas of the prefrontal cortex (PFC), including the dorso- and ventrolateral, and orbital PFC. This study was one of the first to look at the distribution of *HMGN1* expression in the DS brain and demonstrate differences in specific areas.

In summary, the studies mentioned above describe a strong concordance between the increased mRNA and protein levels of HMGN1 and trisomy. This was measured across different mouse models as well as human tissue and cellular systems (Table 4).

**Table 4:** Levels of HMGN1 expression in different mouse models and cell lines

Mouse model	Triplicated regions/genes	Expression of HMGN1	References
Ts16	Harbors triplication of Mmu16	1.6- fold increase in *HMGN1* mRNA in the brain and a 3.3-fold increase in the whole trunk of fetuses	Pash et al. [192]
Ts65Dn	Contains a fusion of the region of Mmu16 orthologous to Hsa21 and the centromeric region of Mmu17, containing a segment of non-DS-related genes	Increase in *HMGN1* mRNA expression found in E15.5 embryonic forebrain tissue	Guedj et al. [197]
Ts1Cje	Harbors partial triplication of Mmu16 (Sod1-Zbtb21 region)	Analysis of raw gene expression data from the Gene Expression Omnibus for Ts1Cje and euploid cerebellar tissue revealed upregulation of *HMGN1* mRNA	Kong et al. [201]
		*HMGN1* was identified as one of 18 differentially expressed genes in the cerebellum of Ts1Cje mice versus disomic mice	Ling et al. [194]
		*HMGN1* mRNA increase in the cerebellum at P0, P15, and P30	Potier et al. [195]
Dp1Tyb	Contains an additional copy of 63% of Mmu16 genes, orthologous to HSA21 genes	Increased *HMGN1* mRNA expression in the hippocampus of adult mice	Lana-Elola et al. [199]
TcMAC21	Incorporates 93% of HSA21 protein coding genes as a separate chromosome	Increase in *HMGN1* mRNA in forebrain tissue at P1	Kazuki et al. [200]
Gene expression omnibus of the national center for biotechnology information data from human DS brain tissue	Triplication of HSA21	*HMGN1* mRNA overexpressed in the hippocampus, cerebellar cortex, primary visual cortex, and pre-frontal cortex	Rodriguez-Ortiz et al. [203]
		*HMGN1* mRNA upregulated 1.5 times in human DS tissue	Kong et al. [201]
Human DS fibroblasts	Triplication of HSA21	1.6 fold increase in *HMGN1* mRNA and 1.8 fold increase in *HMGN1* protein levels in human DS fibroblasts	Pash et al. [193]
Post-mortem DS tissue	Triplication of HSA21	1.6-fold increase in *HMGN1* mRNA expression in the cerebellum and dorsal prefrontal cortex across multiple developmental periods	Olmos-Serrano et al. [202]

Summary of the relevant studies discussed in this review

### DS phenotypes and possible correlation with gene dosage effect of HMGN1

#### Neurodevelopmental phenotypes and HMGN1

Although altered HMGN1 expression has been linked to neurological disorders [180], it is not clear whether the neurological phenotypes seen in DS individuals are caused by elevated HMGN1 levels. Though a direct connection remains elusive, there is a strong basis to suggest that the pathological cellular phenotypes in DS can be associated with enhanced HMGN1 expression in DS brain cells.

Individuals with DS have an 18% reduction in brain volume as compared to those without DS, and this decrease can be detected as early as the second trimester [8, 204, 205]. This is recapitulated in the iPSC-based three-dimensional models (organoids and spheroids), which show a decrease in the size of DS-derived cortical organoids [206] and in our own work utilizing cortical spheroids [207] as well as in the Ts65Dn mouse model [208]. It is noteworthy that in the mouse and human cerebellum, there is also a decrease in the number of granule and Purkinje cells [10, 200, 209, 210]. For the updated and comprehensive review describing DS-related developmental brain changes across different mouse models and cellular systems, please refer to Klein and Haydar [211].

The diminished brain volume in DS is thought to be related to hypocellularity, as reductions in neuronal numbers have been found in multiple areas of the brain, including the neocortex, hippocampus, dentate gyrus, inferior temporal gyrus, fusiform gyrus and cerebellum [9, 210, 212–214]. The hypocellularity of the DS brain is often attributed to the decrease in proliferating NPCs observed as early as 17–21 gestational weeks in human brain tissue [9, 10, 15, 215, 216] and is replicated in NPCs generated from DS-derived iPSCs [206, 217]. Deranged neurogenesis is considered to be a significant factor in the reduced number of neurons in DS brains, as it has been observed both during human fetal development in DS and DS animal models [9, 218], accompanied by delayed cortical lamination and aberrant axonal and dendritic arborization [16], as well as abnormal synaptogenesis [12–14, 16].

Many of these abnormal cellular phenotypes have been linked to other HSA21 genes, such as *DYRK1A* [219], *amyloid precursor protein* (*APP*) [220–224], *DS cell adhesion molecule* (*DSCAM*), *S100 Calcium Binding Protein B* (*S100B*) [225–227], *OLIG2* [228–230], and more. However, it is possible that similar neurodevelopmental and neurodegenerative phenotypes result from the triplication of different genes on HSA21. For example, the enhanced production of APP and the products of its cleavage were shown to induce transcriptomic changes through deficient retrograde trafficking of neurotrophins (mediated through activation of Rab proteins) [220, 221], and to attenuate signaling mediated via Sonic hedgehog (SHH) [222–224]. The triplication of *APP* and *DYRK1A* in DS was also shown to interrupt Notch-mediated signaling [219, 222, 231]. The dysregulation of these signaling pathways has been implicated in abnormalities in neurogenesis [232], neuronal migration [233], and cortical lamination [234]. Importantly, many of the genes driving these pathways are targeted by the PRC2 family members. For example, the *delta-like canonical Notch ligand 3* (*DLL3*) and *basic helix-loop-helix* (*bHLH*) *HES* genes that encode the Notch-HES pathway components are the targets for SUZ-12 binding [68, 80] and are affected by EZH2 knockdown [235], and some of the RAB genes have binding sites for the PRC2 proteins [68, 235, 236].

Similarly, HSA21 genes *DSCAM* and *DYRK1A* have been implicated in the dysregulated neuronal migration and cortical lamination in DS [206, 219]. Deficient neuronal motility and the dysregulation of the “roundabout” signaling pathway have been widely observed in DS [176, 207, 237]. Importantly, genes, such as *ROBO2, ROBO4,* and *SLIT2*, which mediate neuronal migration and axon guidance, appear to be targeted by EZH2 and SUZ-12 [68, 235, 236]. This suggests that triplicated *HMGN1* can lead to developmental phenotypes similar to those induced by other triplicated genes, such as but not limited to *APP*, *DSCAM*, and *DYRK1A*. While direct evidence for the causal link between the triplication of *HMGN1* and the DS-associated cellular processes has not yet been found, the tight regulation of these processes by the members of PRC2 and the global effect of HMGN1 on PRC2 target genes’ transcription and H3K27me3 levels could be the potential missing link.

The data related to the underproduction or overproduction of the inhibitory neurons in DS is controversial, however, the reports related to this disbalance further expose the disturbances in the acquisition of neuronal identity. Several studies have found a decrease in excitatory synapses [238] and an increase in cortical and hippocampal inhibitory synapses in mouse models of DS [239, 240]. Ts65Dn mice have decreased excitatory neuronal density in the neocortex noticeable by P8, and an increase in parvalbumin-(Pvalb) and somatostatin-(SST) positive inhibitory neurons at the same time frame in both the neocortex and the CA1 region of the hippocampus [228]. These disruptions were attributed to the increased dosage effect of *Olig2* in trisomy [228–230], as normalizing *Olig2* expression to euploid levels was enough to rescue the phenotype and reduce the number of Pvalb+ and SST+ interneurons generated in the mouse medial ganglionic eminence (MGE). Similar findings were described in DS iPSC-derived organoids, showing a significant overabundance of the GABAergic neurons and dysregulation of genes related to the establishment of interneuron fate and migration [230]. Other studies, however, found fewer cortical interneurons in DS fetal tissue and adult DS cortex [241]. The underproduction of GABAergic neurons and their abnormal migration was also demonstrated in an in vitro model derived from DS iPSCs and attributed to the increased levels of p21-activated kinase 1 (PAK1) [242], a binding target for SUZ12 [68]. Moreover, the commitment and lineage development of inhibitory neurons are PRC2-dependent [132, 243] and while *OLIG2* is triplicated in DS, it is also known to be highly regulated by HMGN1 [121], suggesting that despite the above discrepancy between the studies, there is a consistency in reported deficits in the interneuron motility and the disbalance between the excitatory and inhibitory neurons that can be potentially caused by the gene dosage of *HMGN1* and subsequent dysregulation of PRC2 targets.

Collectively, neuronal perturbations observed in DS are manifested in hypocellularity, delayed cortical lamination, diminished axonal and dendritic arborization, deficient neurogenesis and synaptogenesis, and disbalance between the inhibitory and excitatory neurons. Extensive research linked these alterations to the gene dosage effect of different triplicated genes or their combinations, as well as to the global genomic and epigenetic dysregulation in DS, as shown in Fig. 1. In this section, we provided evidence that many of the genes coding for the signaling molecules involved in intracellular and intercellular communication, such as SHH, Notch-HES, and “roundabout”, are targets for the transcriptional repression by PRC2 and therefore can be dysregulated due to antagonizing the action of HMGN1 on PRC2 target genes. We also pointed out an additional level of complexity arising from the fact that increased levels of HMGN1 can further potentiate the expression of other HSA21 genes, such as *OLIG1*, *OLIG2,* and *APP*, amplifying the deranged neuronal biology in trisomy.

#### Neuronal cell death in DS and a possible link to PRC2 dysregulation

An enhanced rate of apoptosis has also been found in certain regions of the DS brain, including the hippocampus [9], cerebral cortex [14, 244], cerebellum [244], as well as in the DS iPSC-derived NPCs [217], and cortical spheroids [207]. Increased neuronal cell death was detected in DS fetal brain [244] and the brains of middle-aged DS individuals [245]. However, the extent to which cell death contributes to the overall reduction in the number of neurons remains contested, and evidence related to the role of apoptosis and neurodegeneration in DS is somewhat controversial [10, 218, 227]. The recently demonstrated inhibition of neurodegenerative genes by PRC2 and antagonism between PRC2 and HMGN1 can potentially contribute to the upregulation of the pro-apoptotic genes in DS and enhanced neurodegeneration. The pro-apoptotic gene upregulation in DS has not been previously attributed to the triplication of *HMGN1*.

#### Glial cells in DS and the possible link to the increased gene dosage of HMGN1

In addition to the abnormal proliferation of NPCs, aberrant neurogenesis, and increased cell death, the reduction in neuronal numbers detected in DS can also be causatively linked to an aberrant shift from neurogenesis to gliogenesis [9, 227, 246]. Significantly more astrocytes were detected in human DS frontal lobe brain tissue compared to samples from age-matched controls across different developmental time points [247]. Similar findings have been noted in the hippocampus and frontal cortex of individuals with DS [226]. These findings were attributed previously to the gene dosage effect of the triplicated genes, such as DYRK1A acting through the enhanced STAT activity [248], APP leading to decreased SHH signaling [249], and more.

Since increased levels of HMGN1 can intensify the generation of astrocytes at the late embryonic neocortex at the expense of neurogenesis [179], one can speculate that the gene dosage effect of *HMGN1* in trisomy can be another contributing factor to the gliogenic shift in DS. This is further supported by the regulatory role of the PRC2-deposited H3K27me3 chromatin mark in defining cell identity during the shift from neurogenesis to gliogenesis [134, 159]. Furthermore, enhanced astrocyte production and diminished generation of oligodendrocytes in DS reflect the aberrant transition from astrogenesis to oligodendrogenesis, as has been described previously [222] (Fig. 1).

Indeed, impaired development of oligodendrocytes and aberrant patterns of myelination in DS are well-documented [250]. Children with DS exhibit delayed myelination in the frontotemporal lobes from 2 months to 6 years old, developmentally lagging age-matched controls by almost 12 months. They also display specific delays in myelination in the hippocampus [250, 251]. Overall, people with DS have a decreased density of myelinated fibers and lower levels of myelin basic protein (MBP) and myelin-associated glycoprotein (MAG) [202, 250]. Due to the necessity of myelination for the appropriate and efficient signal propagation, these findings may be significant for explaining the intellectual deficits seen in DS.

Relevant to our studies, PRC2 deficiency is associated with impaired progression from OPCs to myelinating OLs [160]. Even though the dysregulation of the SHH pathway and enhanced expression of the PTCH1 receptor in DS [209, 223, 252, 253] has been attributed, at least partially, to the triplication of *APP* [252], both *SHH* and *PTCH1* are targets for transcriptional repression by PRC2 members [68, 236], and dysregulation of the SHH pathway can be caused by increased transcription of key pathway genes due to the increased dosage of *HMGN1* and alleviation of PRC2 repression. Similarly to neuronal dysregulation, the perturbation in glial cells and diminished maturation of oligodendrocytes can be potentially linked to a deficient silencing of the genes necessary for proper lineage development and functional maturity.

#### Neuroinflammation in DS and HMGN1 gene dosage effect

The role of neuroinflammation in DS has been widely acknowledged. Previous studies demonstrated increased activation of microglia, astrogliosis, dysregulation of inflammation-related genes, as well as the enhanced formation of complement complexes detected in DS brain tissue [189, 254–257]. The frontal cortex of young DS individuals displays an enhanced neuroinflammatory signature of microglia represented by a high ratio of the soma size to the length of the processes and the appearance of the pathological rod-like microglial phenotypes accompanied by enhanced production of pro-inflammatory cytokines [255]. Remarkably, these inflammation-related changes precede the fully blown AD pathology in DS persons [255].

In addition to the increased generation of astrocytes at the expanse of neurons (aka gliogenic shift, as we mentioned earlier), there is an abnormal astrocyte biology. Impaired Ca^2+^ homeostasis and signaling [258], and mitochondrial dysfunction, including altered mitochondrial membrane potential, have been demonstrated in DS-derived astrocytes [259, 260]. In fact, mitochondrial networks are more fragmented and are associated with increased production of reactive oxygen species (ROS) in trisomic astrocytes leading to enhanced oxidative stress and neuronal injury in DS [260]. In addition to the gene dosage effect of APP, DYRK1A, and S100B, implicated previously in enhanced gliosis in DS, other HSA21 genes can play a role in neuroinflammation associated with trisomy. Among these genes are *ubiquitin-specific protease 25* (*USP25*), which regulates microglial activation, *T-cell lymphoma invasion and metastasis 1* (*TIAM1*), and *Superoxide dismutase 1* (*SOD1*), which both participate in a response to the ROS, as well as the interferon family members: *interferon α, β, and ω receptor 1* (*IFNAR1*), *IFNAR2, interferon γ receptor 2* (*IFNGR2*), and *interleukin-10 receptor subunit beta* (*IL10RB*) [261, 262]. Altogether, an increase in the products of these genes can contribute to the neuroinflammatory signature observed in DS. For instance, overexpression of APP and S100B results in an enhanced expression of *interleukin 1* (*IL-1*) and induction of astrocytic and microglial activation [257, 261, 263].

Notably, recent studies showed that aberrant PRC2 activity related to the HMGN1 gene dosage effect could further exacerbate the inflammatory profile associated with trisomy. It was shown that *IFN* signaling is a target of EZH2 suppression in cancer: EZH2 represses IFNγ target genes in prostate [264], colorectal [265], and melanoma [266] cancer cells, while pharmacological inhibition of EZH2 in melanoma results in the upregulated profile of IFNγ and IFN-α downstream targets [266]. In addition, previous studies have linked HMGN1 to a variety of inflammation-related processes [267–269] that have been harnessed for anti-cancer therapeutic approaches. These studies showed that HMGN1 activates and promotes the migration of dendritic cells, participates in an antigen-specific immune response, positively regulates CD8^+ ^T cell activation, and acts as an alarmin [269–271]. On the other hand, the ability of HMGN1 to potentiate the immune response can further contribute to the neuroinflammatory phenotypes seen in DS that can further aggravate the development of AD pathology.

#### Alzheimer’s disease (AD)-related pathology in DS (DS-AD) and HMGN1

Premature development of AD pathology is common in DS, with most individuals displaying AD-related pathology, such as amyloid-β (Aβ) plaques, accumulation of hyper-phosphorylated tau (p-tau), markers of oxidative stress, neuroinflammation, and neurodegeneration by age 40 [257, 261, 263, 272–274]. The density of plaques and tangles in DS-AD is higher compared to that seen in non-DS AD, but the arrangement is similar [275]. There is variability in the age of onset and the level of dementia, despite the near guarantee of someone with DS developing AD during their lifetime [276].

The data related to post-translational histone modifications in AD show patterns of altered histone acetylation, methylation, and phosphorylation [277, 278]. The available information supporting the role of *HMGN1* in DS-AD through the decrease in H3K27me3 and increase in H3K27ac is somewhat controversial since some studies of human postmortem AD brain tissue show different H3K27 modification and based on our knowledge, no genome-wide studies focused on H3K27 marks in DS-AD human brains were performed. However, the existing data obtained from human post-mortem AD brain tissue highlights epigenetic dysregulation in AD pathology and implicates changes in both acetylation and trimethylation histone marks in the disease. Decreased levels of histone H3 (K18/K23) acetylation have been found in the temporal lobe of post-mortem human AD tissue [279]. A recent study, looking specifically at the genome-wide distribution of H3 marks in the entorhinal cortex of post-mortem human tissue from severe cases of AD identified diminished levels of H3K4me3 and enhanced levels of H3K27me3 in association with the disease that was more prominent in males [280]. A genome-wide acetylome study in the human entorhinal cortex of AD patients uncovered a redistributed pattern of H3K27 acetylation and identified 4,162 differentially modified peaks (both hypo- and hyper-acetylated) as compared to matched controls [281]. The investigators found alterations in the H3K27ac patterns near the following genes involved in the pathological generation of Aβ: *APP*, *microtubule-associated protein tau* (*MAPT*), *presenilin-1* (*PSEN1*), and *PSEN2* [282–285]. Gene ontology analysis revealed that hyperacetylated genes were enriched in categories associated with Aβ metabolic processes and tau interactome, while hypoacetylated genes were enriched in categories associated with neurotransmitter function, neuronal transmission, and synapses.

Additionally, increased levels of acetylated histone H3 and H4 were found in the pyramidal neurons in the inferior temporal gyrus of post-mortem AD tissue and were significantly correlated with both tau and β-amyloid load [286]. A recent multiome study further confirmed the previously shown reconfiguration of acetylation patterns in the lateral temporal lobe from AD patients and revealed an increase in H3K27ac and H3K9ac marks [287]. Remarkably, the gain of these acetylation marks was linked to disease-specific AD pathways, and increasing H3K27ac and H3K9ac levels in *Drosophila,* performed by the same group, exacerbated Aβ42-induced neurodegeneration.

Altogether, these studies show the functional relevance between the posttranslational modification of histones and the classical features of AD pathology in mouse and human brains. The changes in the epigenetic landscape induced by HMGN1 in DS may contribute to the development of AD pathology. Notably, a meta-analysis of cognition-related genome-wide association studies yielded *HMGN1* as only one significantly associated gene [288]. Both AD and DS pathology have been connected to changes at H3K27 acetylation/trimethylation marks but future studies are needed to provide a causative link between the specific changes to the epigenetic environment induced by overexpression of *HMGN1* in DS and the development of AD pathology.

## Conclusion

**Fig. 2:**
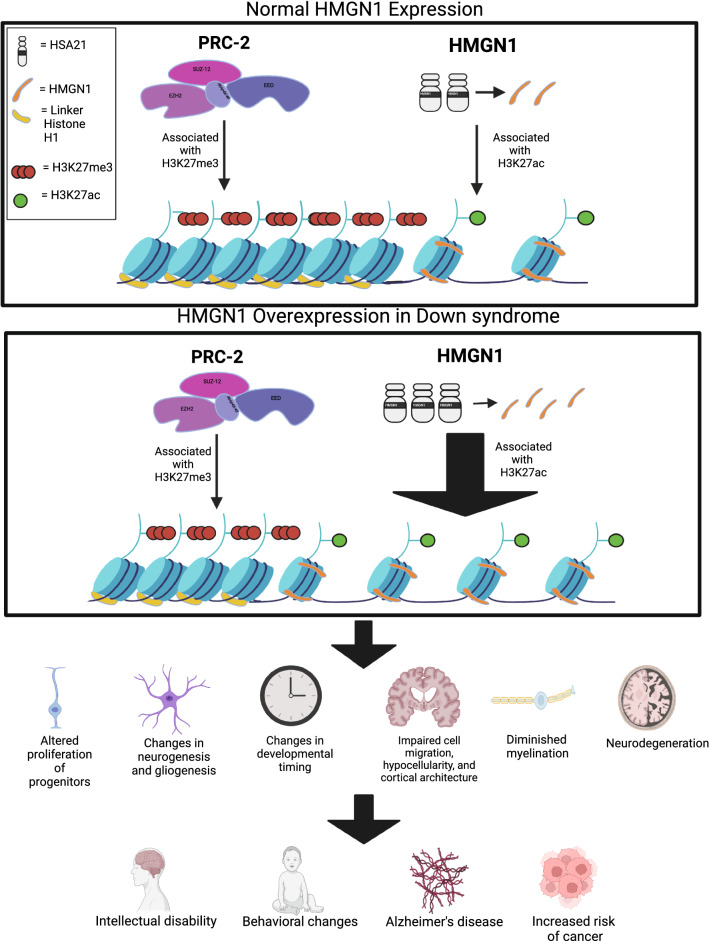
Schematic illustrating the effects of increased dosage of HMGN1 in DS through the antagonizing action on PRC2. The epigenetic changes induced by increased levels of PRC2 lead to diverse transcriptomic and phenotypic changes that can potentially lead to the pathological features observed in DS. Created with BioRender.com

In this review, we discussed a possible mechanistic connection between the *HMGN1* triplication in DS and downstream events leading the neurodevelopmental and neurodegenerative pathology in trisomy. We specifically hypothesized that increased *HMGN1* dosage promotes global chromatin accessibility for transcription, thus leading to the derepression of PRC2 target genes, causatively linking the triplication of *HMGN1* in trisomy to several DS-related cellular phenotypes. We present a summary of this hypothesis in Fig. 2.

## Data Availability

Not applicable.

## Abbreviations

DS: Down syndrome
IQ: Intelligence quotient
B-ALL: B-cell acute lymphoblastic leukemia
HMGN1: High mobility group binding domain 1
DYRK1A: Dual specificity tyrosine-phosphorylation-regulated-kinase 1A
ETS2: ETS proto-oncogene 2
BRWD1: Bromodomain and WD repeat domain containing 1
RUNX1: Runt-related transcription factor 1
DNMT3L: DNA (cytosine-5)-metyltransferase 3-like
SON: SON RNA and DNA binding protein
PRC2: Polycomb repressive complex 2
HAT: Histone acetyltransferase
HDAC: Histone deacetylase
ChIP: Chromatin immunoprecipitation
TSS: Transcription start site
HMG: High mobility group
CHUD: Chromatin unfolding domain
MEF: Mouse embryonic fibroblast
ESC: Embryonic stem cell
NBD: Nuclear-binding domain
DKO: Double-knock-out
HMT: Histone methyltransferase
EED: Embryonic ectoderm development
EZH2: Enhancer of zeste
SUZ-12: Suppressor of zeste
BrdU: Bromodeoxyuridine
DG: Dentate gyrus
SVZ: Subventricular zone
DA: Dopamine
5HT: Serotonin
MSN: Medium spiny neuron
IUE: Intra-utero electroporation
shRNA: Short-hairpin RNA
CP: Cortical plate
OPC: Oligodendrocyte precursor cell
NSC: Neural stem cell
Olig2: Oligodendrocyte transcription factor 2
MBP: Myelin basic protein
iPSC: Induced pluripotent stem cell
PolII: RNA polymerase II
Pdgfr-α: Platelet-derived growth factor receptor alpha
MNase: Micrococcal nuclease
TC-rMNase-seq: Time-course digestion with reduced MNase levels followed by high-throughput sequencing
Camk2a: Calcium/calmodulin dependent protein kinase II alpha
Pmaip1: Phorbol-12-myristate-13-acetate-induced protein 1
BID: BH3 interacting domain death agonist
Noxa: NADPH oxidase activator 1
Cdkn2a/b: Cyclin-dependent kinase inhibitor 2A/B
Igfbp3: Insulin-like growth factor-binding protein 3
CRLF2: Cytokine receptor-like factor 2
Mmu16: Mouse chromosome 16
ATAC-seq: Transposase-accessible chromatin sequencing
EB: Embryoid body
CNPase: 2ʹ,3ʹ-Cyclic-nucleotide 3ʹ-phosphodiesterase
PLP: Proteolipid protein
MeCP2: Methyl CpG-binding protein 2
GEO: Gene expression omnibus
PFC: Prefrontal cortex
APP: Amyloid precursor protein
DSCAM: DS cell adhesin molecule
SHH: Sonic hedgehog
bHLH: Basic helix-loop-helix
Pvalb: Paralbumin
Sst: Somatostatin
MGE: Medial ganglionic eminence
PAK1: P21 activated kinase 1
MAPT: Microtubule-associated protein tau
PSEN1: Presenilin-1
JAK-STAT: Janus tyrosine kinase-signal transducer and activator of transcription
